# hsa-miR-34a-5p enhances temozolomide anti-tumoral effects on glioblastoma: in-silico and in-vitro study

**DOI:** 10.17179/excli2023-6404

**Published:** 2024-03-13

**Authors:** Mahdi Abdoli Shadbad, Amir Baghbanzadeh, Behzad Baradaran

**Affiliations:** 1 Student Research Committee, Tabriz University of Medical Sciences, Tabriz, Iran; 2 Immunology Research Center, Tabriz University of Medical Sciences, Tabriz, Iran; 3 Department of Immunology, Faculty of Medicine, Tabriz University of Medical Sciences, Tabriz, Iran

**Keywords:** hsa-miR-34a-5p, temozolomide, glioblastoma, MAPK pathway, single-cell RNA sequencing

## Abstract

Glioblastoma multiform (GBM) is a commonly diagnosed brain neoplasm with a poor prognosis. Accumulating evidence has highlighted the significance of microRNA (miR) dysregulation in tumor development and progression. This study investigated the effect of hsa-miR-34a-5p and its combination with temozolomide on GBM, the related molecular mechanisms, and the signaling pathway using *in-silico* and *in-vitro* approaches. The *in-silico* tumor bulk and single-cell RNA sequencing analyses were done on TCGA-GTEx, CGGA, GSE13276, GSE90603, and GSE182109 datasets. After selecting the A172 cell line, hsa-miR-34a-5p mimics were transfected, and the cell viability, migration, cell cycle, clonogenicity, and apoptosis of studied groups were studied using MTT, scratch, flow cytometry, colony formation, and Annexin V/PI assays. The mRNA expression of *CASP9*, *CASP3*, *CASP8*, *MMP2*, *CD44*, *CDK6*, *CDK4*, *CCND1*, *RAF1*, *MAP2K1*, *MET*, *SRC*, and *CD274* was studied using qRT-PCR method. hsa-miR-34a-5p downregulated *RAF1 *expression, as the signaling factor of the MAPK pathway. The combined treatment significantly downregulated the expression of *MET*, *SRC*, and *MAP2K1*, leading to the inhibition of the MET/MAPK pathway compared to temozolomide. Besides exerting anti-tumoral effects on the cell viability, migration, cell cycle, apoptosis, and clonogenicity of A172 cells, its combination with temozolomide enhanced temozolomide anti-tumoral effect. Compared to temozolomide, the combined treatment significantly decreased *CDK4*, *CDK6*, *CCND1*, and *MMP2* expression. hsa-miR-34a-5p targets *RAF1*, as the signaling factor of the MAPK pathway, and potentiates the temozolomide anti-tumoral effect on A172 cells.

**Table 1:** The primer sequence used for qRT-PCR

Gene		Sequence
Caspase 3	Forward	CAAACCTCAGGGAAACATTCAG
	Reverse	CACACAAACAAAACTGCTCC
Caspase 8	Forward	CGGACTCTCCAAGAGAACAGG
	Reverse	TCAAAGGTCGTGGTCAAAGCC
Caspase 9	Forward	CTGTCTACGGCACAGATGGAT
	Reverse	GGGACTCGTCTTCAGGGGAA
CD44	Forward	CTGCCGCTTTGCAGGTGTA
	Reverse	CATTGTGGGCAAGGTGCTATT
CDK6	Forward	GTCTGATTACCTGCTCCGCGA
	Reverse	CGAAGCGAAGTCCTCAACACA
CDK4	Forward	CCATCAGCACAGTTCGTGAGGT
	Reverse	TCAGTTCGGGATGTGGCACAGA
CCND1	Forward	CCCTCGGTGTCCTACTTCAA
	Reverse	GTGTTCAATGAAATCGTGCG
RAF1	Forward	AGTCACAGCGAATCAGCCTC
	Reverse	GCCTAATTTTGTTTTTCTCCTGGG
MAP2K1	Forward	CAATGGCGGTGTGGTGTTC
	Reverse	GATTGCGGGTTTGATCTCCAG
MET	Forward	GGACATCAGAGGGTCGCTTCA
	Reverse	TCTGGAGACACTGGATGGGAGT
SRC	Forward	TCGTGCGAGAAAGTGAGACC
	Reverse	GCTTGCGGATCTTGTAGTGC
CD274	Forward	TGCCGACTACAAGCGAATTACTG
	Reverse	CTGCTTGTCCAGATGACTTCGG
MMP2	Forward	AGCGAGTGGATGCCGCCTTTAA
	Reverse	CATTCCAGGCATCTGCGATGAG
18s	Forward	ACCCGTTGAACCCCATTCGTGA
	Reverse	GCCTCACTAAACCATCCAATCGG

## Introduction

Glioblastoma multiform (GBM) is one of the commonly diagnosed primary brain malignancies with an extremely poor prognosis; the highly aggressive nature of GBM, tumor heterogeneity, and chemoradioresistance have been introduced as the culprits for the poor prognoses of affected patients (Abdoli Shadbad et al., 2021[3]). Although a recent double-blind, phase 3 trial has provided promising results about the therapeutic significance of vorasidenib in patients with low-grade glioma IDH1- or IDH2-mutant, temozolomide resistance is still a worrisome concern for GBM patients (Mellinghoff et al., 2023[31]). Therefore, there is an urgent need to identify novel treatments for affected patients.

As small non-coding RNAs, microRNAs (miRs) have considerable roles in regulating the expression of specific genes both directly and indirectly (Dastmalchi et al., 2021[9]). The RNA polymerase II-mediated pri-miR transcription usually begins the biosynthesis of mature miRs (Peng and Croce, 2016[34]). Then, pri-miRs are cleaved into approximately 85 nucleotide miRs, known as pre-miRs. Following RNase III enzyme Dicer-mediated processing of pre-miRs and duplex unwinding, mature miRs can be incorporated into RNA-induced silencing complex (RISC) for targeting the expression of specific mRNAs (Borchert et al., 2006[7]). It has become a well-established fact that dysregulated expression of miRs is among the culprits for developing and progressing various malignancies; the tumor-suppressive miRs downregulation and oncogenic miRs upregulation increases oncogenes' expression and decreases tumor-suppressive genes' expression (Shadbad et al., 2022[36]). Therefore, identifying the role of miRs in the development of various cancers and altering the dysregulated expression of miRs can be considered a therapeutic approach for treating cancers like GBM. 

The miR-34 family has three members, i.e., miR-34a, miR-34b, and miR-34c; the chromosomal locus of miR-34a differs from the miR-34b/c (Wong et al., 2011[42]). In mice, miR-34a is mainly expressed in the brain, and miR-34b/c is predominantly expressed in the lung (Hermeking, 2010[17]). Following DNA damage, P53 activates the expression of mature miR-34 members to inhibit cell replications (Hermeking, 2010[17]). It has been reported that miR-34a expression is suppressed in various malignant cell lines, like pancreatic adenocarcinoma, prostate carcinoma, colon adenocarcinoma, melanoma, and breast adenocarcinoma (Lodygin et al., 2008[26]). Multiple studies have reported the anti-tumoral effect of miR-34a-5p ectopic expression on the development of various cancers, like breast adenocarcinoma, non-small cell carcinoma, and glioma (Li et al., 2020[22], Luo et al., 2020[27], Noorolyai et al., 2022[32], Shadbad et al., 2021[37]). However, there is no study investigating the molecular mechanism of hsa-miR-34a-5p ectopic expression on GBM development and chemosensitivity. 

The current study aimed to study the effect of hsa-miR-34a-5p ectopic expression on GBM development and temozolomide sensitivity in A172 cells. Besides deep *in-silico* studies, *in-vitro* studies were performed to better understand the effect of the hsa-miR-34a-5p ectopic expression on the intracellular signaling pathway of GBM.

## Method and Materials

### In-silico investigation

#### Bioinformatic tools

The MIENTURNET database (http://userver.bio.uniroma1.it/apps/mienturnet/) was used to identify miR gene interactions (Licursi et al., 2019[25]). Cytoscape was used to have access to GeneMANIA and STRING datasets for studying the interactions between the studied genes (Shannon et al., 2003[38]). The interaction map between hsa-miR-34a-5p and *RAF1* was obtained from the MIRmap database (Vejnar and Zdobnov, 2012[40]). The correlational study between hsa-miR-34a-5p and *RAF1* was done based on the TCGA-GBM dataset from cBioPortal, and the prognostic studies were conducted based on the CGGA dataset (Cerami et al., 2012[8]; Zhao et al., 2021[44]). The CCLE dataset was accessed to investigate the mRNA expression of *RAF1* and miR-34a in U87MG and A172 cell lines (Ghandi et al., 2019[14]). The TCGA-GTEx dataset was accessed using the GEPIA 2 (http://gepia2.cancer-pku.cn/) to study *RAF1* expression in GBM and non-malignant brain samples (Tang et al., 2019[39]).

#### Tumor bulk RNA analysis

We chose the GSE13276 and GSE90603 datasets from the Gene Expression Omnibus (GEO) database of NCBI to examine the mRNA and miR expression in GBM tissues and surrounding non-tumoral tissues. GSE13276 and GSE90603 utilized [HG-U133A] Affymetrix Human Genome U133A Array and GPL21572 [miRNA-4] Affymetrix Multispecies miRNA-4 Array [ProbeSet ID version] to examine mRNA and miR expression in GBM and surrounding non-malignant tissues, respectively (Gulluoglu et al., 2018[15]; Mangiola et al., 2013[29]). The expression values of the dataset were normalized in a quantile manner, and the subsequent clustering was performed using R software. 

#### Single-cell RNA sequencing

The primary GBM's raw single-cell RNA transcriptomes were made available through the GSE182109 dataset (Abdelfattah et al., 2022[1]). This dataset used GPL20301 Illumina HiSeq 4000 (Homo sapiens) for single-cell RNA sequencing. After choosing 6 samples, the Seurat (version 4.1.0) (Hao et al., 2021[16]) R package was used to analyze raw single-cell RNA sequencing data. For analysis, cells with mitochondrial gene expression of less than 10 % and more than 500 expressed genes were chosen. Following data normalization, finding variable genes, and integrating data from multiple samples, we scaled the data and ran the principal component analysis. The uniform manifold approximation and projection (UMAP) clustering techniques were then used to group the cells into a two-dimensional figure with a resolution of 0.1. To annotate the found clusters, we used the PanglaoDB (Franzén et al., 2019[12]) and CellMarker (Zhang et al., 2019[43]) datasets. 

### In-vitro studies

#### Cell culture

After purchasing the A172 cell line from the cell bank of Pasture Institute (Tehran, Iran), the malignant cells were grown in RPMI-1640 enriched with 10 % fetal bovine serum (GIBCO, Carlsbad, CA) and 1 % penicillin/streptomycin. The cells were incubated at 37 °C in a 95 % humidified, 5 % CO_2_ incubator.

#### Transfecting hsa-miR-34a-5p

After reaching 80 % confluency, the number of malignant cells was counted, and they were suspended in a cold electroporation buffer. 1×10^6^ cells were transfected with scramble, 5, 7.5, and 10 pmol of hsa-miR-34a-5p mimics (UGGCAGUGUCUUAGCUGGUUGU) using a Gene Pulser Electroporation (Bio-Rad, CA, USA) and a 0.4 cm^3^ Gene Pulser Cuvette (Bio-Rad, CA, USA).

#### MTT assay

The half-maximal inhibitory concentration (IC_50_) of temozolomide on A172 cells was studied using the 3-(4,5-dimethylthiazol-2-yl)-2,5-diphenyl-2H-tetrazolium bromide (MTT) assay. 50 μl of MTT (2 mg/ml) was added to each well after seeding 15×10^3^ of A172 cells into a 96-well plate. Then, the cells were incubated for 180 minutes in the incubator. Following removing the medium, 150 μl of dimethyl sulphoxide (DMSO) was added to each well, and the plate was incubated for 30 minutes. After shaking for 10 minutes, the optical density (OD) of each well was assessed at 570 nm using an ELISA reader (Sunrise RC, Tecan, Switzerland).

#### Colony formation assay

The viability and stemness of the A172 cells were investigated using the colony formation assay. After seeding 5×10^3^ transfected and non-transfected cells into a six‐well plate and incubating for 14 days and treatment, the cells were stained using crystal violet. The inverted microscope (Optika, XDS‐3, Italy) was used to obtain microscopic pictures of the colonies.

#### Scratch assay

The scratch assay was used to study the migration of A172 cells. 2 × 10^5^ A172 cells were seeded in a 24-well plate and subjected to treatments. A yellow sterile pipette tip was then used to scrape the cellular monolayer. Using an inverted microscope (Optika, XDS-3, Italy), the associated pictures were taken at 0, 12, 24, and 48 hours after incubation.

#### Annexin V/propidium iodide (PI) assay

The annexin V/PI assay was used to study A172 cell apoptosis. After seeding 2 × 10^5^ transfected and non-transfected A172 cells in a six-well plate and incubating for 24 hours, we treated one group of transfected and one group of non-transfected cells with temozolomide. After another incubation for 24 hours, the cells were stained with annexin V/PI based on the manufacturer's protocols (EXBIO, Vestec, Czech Republic). Then, flow cytometry (MiltenyBiotec™ FACS Quant 10; MiltenyBiotec, Germany) was used to evaluate the apoptosis rate of each group. The data were analyzed using FlowJo software (Tree Star, CA, USA). 

#### Cell cycle assay

The above-mentioned seeding and treatment procedures were used in this assay as well. Single cells from each group were obtained, fixed with 80 % ethanol, and stored at -20 °C for the duration of the next day. After RNase treatment and DAPI staining, cell distribution in the cell cycle phases was studied using flow cytometry (MiltenyBiotecTM FACS Quant 10; MiltenyBiotec, Germany). The data were analyzed using the FlowJo software (Tree Star, CA, USA).

#### qRT-PCR

Total RNA was extracted using the TRIzol reagent (GeneAll Biotechnology, Seoul, South Korea). A spectrophotometer (Thermo Fisher Scientific, Lenexa, South Korea) was used to examine the purity and concentration of the extracted RNA. The complementary DNA (cDNA) was synthesized using a thermal cycler system and the AddScript cDNA Synthesis Kit (ADDBIO, South Korea). Using a StepOnePlusTM Real-Time PCR System (Thermo Fisher, MA, USA), the mRNA expression of *CASP9*, *CASP3*, *CASP8*, *MMP2*, *CD44*, *CDK6*, *CDK4*, *CCND1*, *RAF1*, *MAP2K1*, *MET*, *SRC*, and *CD274* was studied. 18s was used as the housekeeping gene. Before the qRT-PCR, the pair primer sequences were blasted before using the NCBI website (https://www.ncbi.nlm.nih.gov/tools/primer-blast/) (Table 1(Tab. 1)). The obtained ct values were analyzed using log_10_ 2^-∆∆Ct^.

#### Statistical analyses

Utilizing GraphPad Prism V 8.0.2 (GraphPad Software, CA, USA), the *in-vitro* data were analyzed. To conduct statistical comparisons between more than two groups, the one-way ANOVA was used. The Shapiro-Wilk test was used to determine whether the data had a normal distribution or not. P-values less than 0.05 were considered statistically significant.

## Results

### hsa-miR-34a-5p has considerable interaction with studied oncogenes

**Figure 1:**
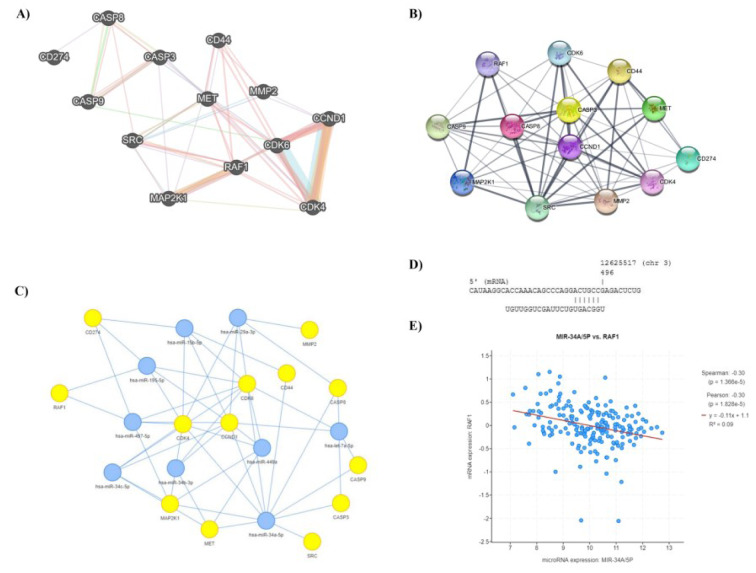
hsa-miR-34a-5p is the candidate tumor-suppressing human miR. A) Gene interaction based on GeneMANIA. B) Interactions between studied genes based on STRING. C) hsa-miR-34a-5p can be the only human miR with interactions with maximum numbers of studied oncogenes, i.e., *MMP2*, *CD44*, *CDK6*, *CDK4*, *CCND1*, *RAF1*, *MAP2K1*, *MET*, *SRC*, and *CD274*. D) hsa-miR-34a-5p has interaction with *RAF1*. E) Based on the cancer genome atlas program (TCGA)-glioblastoma multiforme (GBM) data, there is a significant negative correlation between the expression of hsa-miR-34a-5p with *RAF1* expression in primary GBM tissues.

Based on STRING and GeneMANIA databases, our studied genes, i.e., *CASP9*, *CASP3*, *CASP8*, *MMP2*, *CD44*, *CDK6*, *CDK4*, *CCND1*, *RAF1*, *MAP2K1*, *MET*, *SRC*, and *CD274*, have considerable interactions with each other (Figure 1A, B(Fig. 1)). With a score cut-off of 0.4, the interactions demonstrated by STRING are originated from genomic predictions, high-throughput lab experiments co-expression, automated text-mining, and previous knowledge in databases. With the max resultant attribute of null, GeneMANIA investigates the interactions between genes regarding co-expression, co-localization, pathways, genetic interactions, predicted, physical interactions, and shared protein domains. These results indicated that the studied genes have considerable interactions, and their modulation might affect each other, leading to tumor regression. In the next step, we attempted to identify a miR that has the most interactions with the studied oncogenes, i.e.,* MMP2*, *CD44*, *CDK6*, *CDK4*, *CCND1*, *RAF1*, *MAP2K1*, *MET*, *SRC*, and *CD274*, that have pivotal roles in migration, stemness, cell cycle, and tumor development. For this aim, we accessed the miRTarbase database via the MIENTURNET. It has been found that hsa-miR-34a-5p is the only human miR with interactions with the maximum number of studied oncogenes (Figure 1C(Fig. 1)). Therefore, we selected hsa-miR-34a-5p for the current study. Besides, our study on the miRmap has indicated that hsa-miR-34a-5p can interact with *RAF1 *(Figure 1D(Fig. 1)). Also, our analysis of TCGA-GBM has indicated a significant negative correlation between hsa-miR-34a-5p and *RAF1* expression in primary GBM tissues (r = -0.3 and P-value < 0.05) (Figure 1E(Fig. 1)). Therefore, hsa-miR-34a-5p is selected for our *in-vitro* studies.

### RAF1 is significantly upregulated in primary GBM compared to non-malignant brain tissues

**Figure 2:**
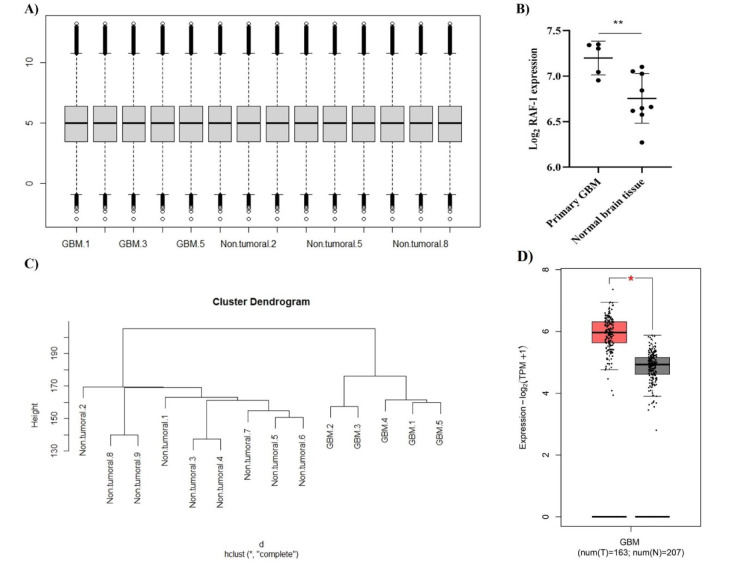
*RAF1* is significantly upregulated in primary glioblastoma multiforme (GBM) tissues compared to normal brain tissues. A) The quantile normalization of included samples of the GSE13276 dataset. The Y-axis denotes the normalized expression level following quantile normalization. B) Based on included samples of GSE13276 datasets, *RAF1* is significantly upregulated in primary GBM tissues compared to normal brain tissues. C) Unsupervised clustering of the included samples. D) Based on GBM-TCGA and genotype-tissue expression (GTEx) samples, *RAF1* is significantly upregulated in primary GBM tissues compared to normal brain tissues. The Y-axis denotes the expression Log_2_ (transcripts per million+1). **: P-value < 0.01

Before analyzing the expression level of *RAF1* in primary GBM tissues and non-tumoral brain tissues, we normalized the expression values of the GSE13276 dataset (Figure 2A(Fig. 2)). We included 5 primary GBM tissues and 9 non-tumoral brain tissues in our analysis; our analyses on GSE13276 have indicated that *RAF1* is significantly upregulated in primary GBM tissues compared to normal brain tissues (P-value = 0.0074) (Figure 2B, C(Fig. 2)). Also, we studied its expression in the primary GBM tissues of TCGA-GBM and normal brain tissues of GTEx. It has been found that *RAF1* is significantly upregulated in primary GBM compared with normal brain tissues (Figure 2D(Fig. 2)). 

### There is no significant difference between hsa-miR-34a-5p expression in GBM and normal brain tissues 

**Figure 3:**
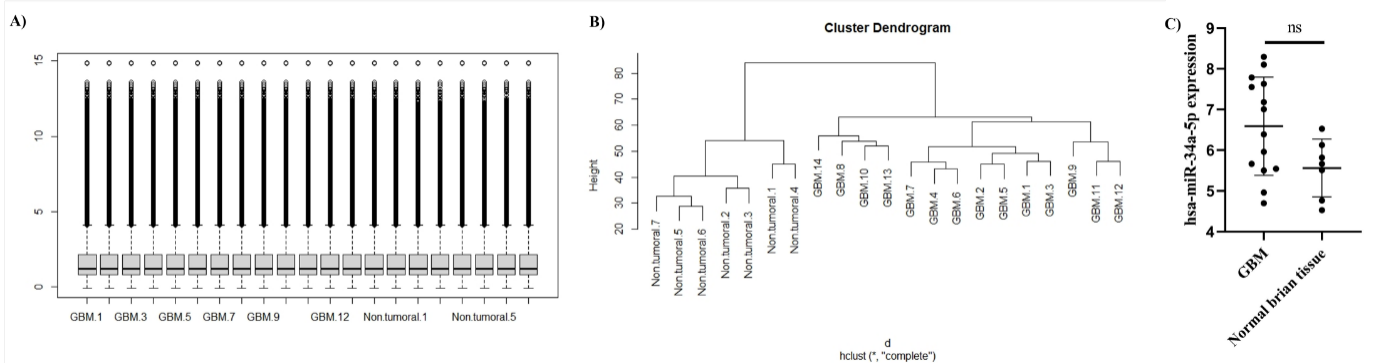
There is no significant difference between hsa-miR-34a-5p expression in glioblastoma multiforme (GBM) and non-tumoral brain samples. A) The quantile normalization of included samples of the GSE90603 dataset. The Y-axis indicates the normalized expression level after quantile normalization. B) Unsupervised clustering of the included samples. C) Based on the included samples of GSE90603 datasets, there is no significant difference between hsa-miR-34a-5p expression in GBM and non-tumoral brain samples.

Then, we studied the expression level of hsa-miR-34a-5p in GBM and non-tumoral brain tissues. For this aim, we normalized the data of the GSE90603 dataset and included 14 GBM tissues and 7 non-tumoral brain samples (Figure 3A, B(Fig. 3)). Our results have shown no statistically significant difference between the expression level of hsa-miR-34a-5p in GBM tissues and normal brain tissues (Figure 3C(Fig. 3)).

### Single-cell RNA sequencing on primary GBM

**Figure 4:**
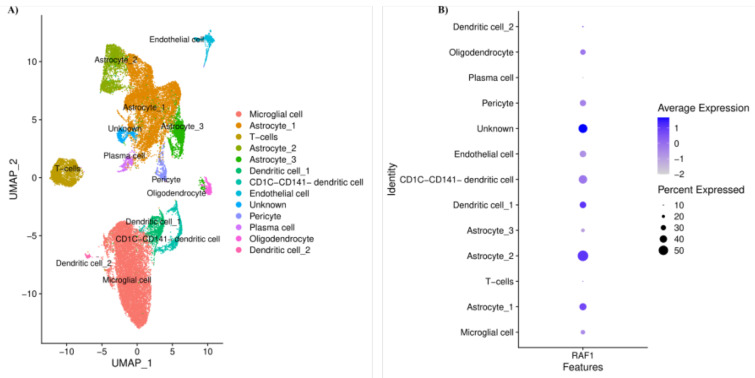
Clustering and cell annotation were applied to identify 13 cell populations in the tumor microenvironment of the included glioblastoma multiforme (GBM) samples

After demonstrating that *RAF1* is significantly upregulated in primary GBM compared to normal brain tissues, we aimed to demonstrate which cell population is responsible for the altered expression of *RAF1* in the primary GBM. After excluding low-quality cells from GBM samples, we included 30,169 cells from patients with GBM in our single-cell RNA sequencing analysis. After-wards, we identified 13 main cell populations in the included GBM microenvironment (Figure 4A(Fig. 4)). Our results have indicated that both tumoral and non-tumoral cells in the tumor microenvironment can be responsible for the altered expression of *RAF1* in GBM. Our results have demonstrated that astrocytes have relatively high expression of *RAF1* in the included GBM samples (Figure 4B(Fig. 4)).

### The prognostic values of hsa-miR-34a-5p and RAF1 in primary GBM patients 

**Figure 5:**
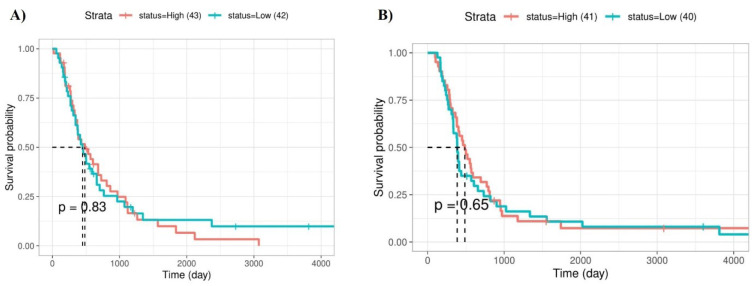
The prognostic values of *RAF1* and hsa-miR-34a-5p in primary glioblastoma multiforme (GBM) patients of the Chinese glioma genome atlas (CGGA) cohort. A) Although there has been a trend in which the increased expression of *RAF1* has been associated with the inferior survival of primary GBM patients, this trend has not been statistically significant. B) There is no statistically significant association between hsa-miR-34a-5p expression and the survival of primary GBM patients.

We investigated the prognostic values of hsa-miR-34a-5p and *RAF1* in patients with primary GBM using the CGGA database. A trend in which increased expression of *RAF1* was linked with worse survival was seen; however, this trend did not amount to statistical significance (P-value = 0.83) (Figure 5A(Fig. 5)). Besides, our results have indicated no statistically significant associations between the survival of primary GBM patients with hsa-miR-34a-5p expression level (P-value = 0.65) (Figure 5B(Fig. 5)). 

### A172 cells have decreased expression of miR-34a and increased expression of RAF1 compared to U87MG cells

**Figure 6:**
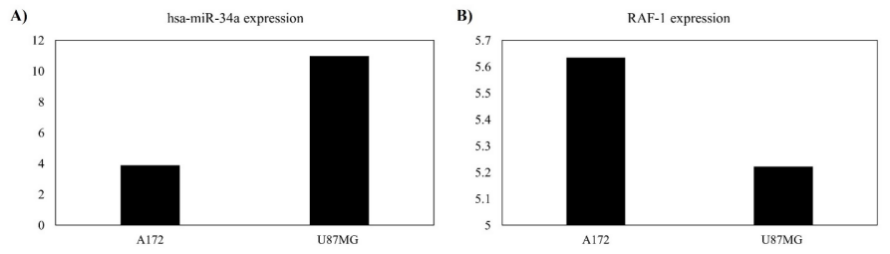
Based on the cancer cell line encyclopedia (CCLE) datasets, A172 cells have decreased expression of miR-34a and increased expression of *RAF1* compared to U87MG cells. A) miR-34a expression in A172 and U87MG cells. The Y-axis denotes the expression levels of miR-34a. B) *RAF1* expression in A172 and U87MG cells. The Y-axis indicates the expression levels of *RAF1*.

We accessed the CCLE dataset to select a cell line with a high expression of *RAF1* and a low expression of miR-34a for our *in-vitro* study. A trend in which A172 cells have decreased expression of miR-34a and increased expression of *RAF1* compared to U87MG cells has been identified (Figure 6A, B(Fig. 6), respectively). Therefore, we selected the A172 cell line for our *in-vitro* studies. 

### hsa-miR-34a-5p ectopic expression has a cytotoxic effect on A172 cells and sensitizes A172 cells to temozolomide

**Figure 7:**
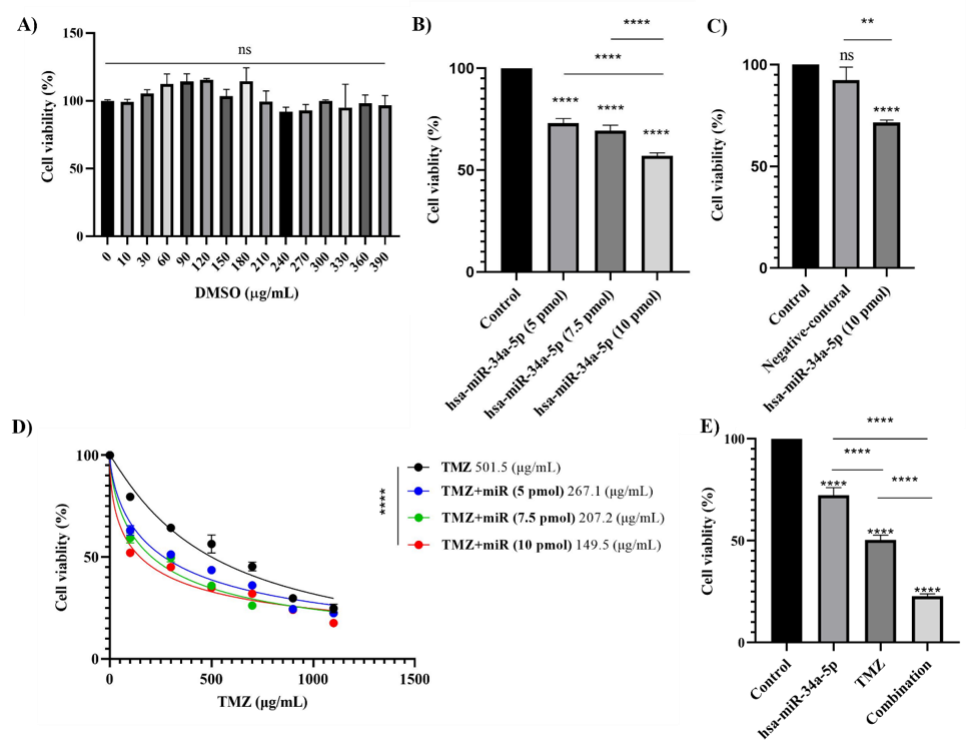
The cytotoxic effect of different doses of hsa-miR-34a-5p on A172 cells. A) Cell culture dimethyl sulfoxide (DMSO) has no significant cytotoxic effect on A172 cells. B) 10 pmol of hsa-miR-34a-5p is the optimal dose for inducing a cytotoxic effect on A172 cells compared to 5 pmol and 7.5 pmol of hsa-miR-34a-5p. C) Transfection procedure has no significant cytotoxic effect on A172 cells. D) The ectopic expression of 10 pmol hsa-miR-34a-5p significantly enhances the chemosensitivity of A172 cells to temozolomide. E) The cytotoxicity of combination therapy is superior to monotherapy with temozolomide and 10 pmol hsa-miR-34a-5p ectopic expression; ns: non-significant, **: P-value≤0.01, and ****: P-value≤0.0001

Since DMSO was used as a solvent for temozolomide, we tested if DMSO had a cytotoxic effect on A172 cells or not. Our results have shown that DMSO has no significant cytotoxic effect on A172 cells (Figure 7A(Fig. 7)). Then, we investigated the effect of hsa-miR-34a-5p transfection with doses of 5 pmol, 7.5 pmol, and 10 pmol on the cell viability of A172 cells. Our results have indicated that 10 pmol is the optimal dose for the hsa-miR-34a-5p ectopic expression-mediated cytotoxicity on A172 cells (Figure 7B(Fig. 7)). We also used scramble miR to exclude the potential cytotoxicity induced in the transfection process. It has been shown that the transfection of negative control miR has no significant cytotoxic effect on A172 cells (Figure 7C(Fig. 7)).

Afterwards, we investigated the effect of the hsa-miR-34a-5p ectopic expression on the chemosensitivity of A172 cells to temozolomide. Our results have indicated that 10 pmol hsa-miR-34a-5p transfection significantly decreases the IC_50_ of A172 cells (P-value < 0.0001) (Figure 7D(Fig. 7)). Consistent with this, we have shown that the combination therapy of temozolomide with ectopic expression of 10 pmol hsa-miR-34a-5p is superior to monotherapy with temozolomide and mono-therapy with 10 pmol hsa-miR-34a-5p ectopic expression in terms of inducing cytotoxicity (both P-values <0.0001) (Figure 7E(Fig. 7)). Therefore, 10 pmol hsa-miR-34a-5p transfection enhances the chemosensitivity of A172 cells to temozolomide.

### hsa-miR-34a-5p ectopic expression increases the anti-tumoral effect of temozolomide on A172 cell migration

**Figure 8:**
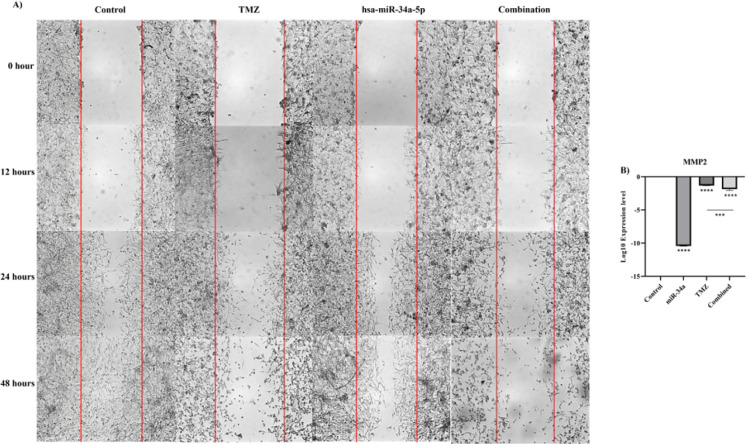
The effect of hsa-miR-34a-5p ectopic expression and temozolomide on the migration of A172 cells. A) Wound-healing assay. B) *MMP2* mRNA expression; ***: P-value≤0.001, and ****: P-value≤0.0001

We performed the wound-healing assay to investigate the effect of hsa-miR-34a-5p ectopic expression and its combination with temozolomide on the migration of A172 cells. Our results have shown that hsa-miR-34a-5p ectopic expression significantly decreases A172 cell migration (Figure 8A(Fig. 8)). Also, hsa-miR-34a-5p significantly increases the anti-tumoral effect of temozolomide on A172 cell migration (Figure 8A(Fig. 8)). Consistent with this, hsa-miR-34a-5p considerably downregulates *MMP2* mRNA expression, and the combined therapy has lower *MMP2* expression compared to temozolomide group (P-value < 0.0001, and P-value = 0.0006, respectively) (Figure 8B(Fig. 8)). Therefore, hsa-miR-34a-5p ectopic expression increases the anti-tumoral effect of temozolomide on A172 cell migration.

### Combined temozolomide therapy with hsa-miR-34a-5p increases temozolomide-mediated apoptosis on A172 cells

**Figure 9:**
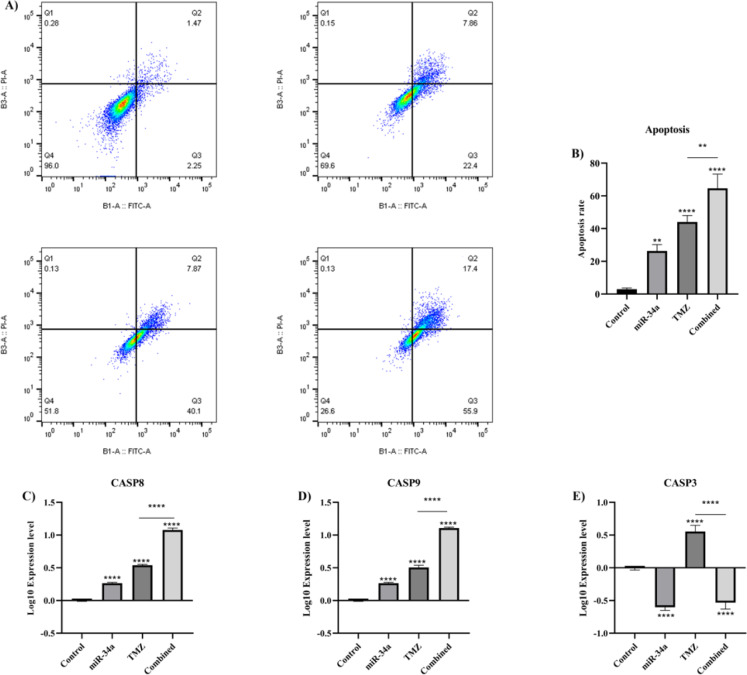
The effect of hsa-miR-34a-5p ectopic expression and temozolomide on the apoptosis of A172 cells. A) Annexin V/propidium iodide (PI) assay. B) Apoptosis rate comparison between groups. C) *CASP8* mRNA expression. D) *CASP9* mRNA expression. E) *CASP3* mRNA expression. **: P-value≤0.01, and ****: P-value≤0.0001

Then, we aimed to study the effect of hsa-miR-34a-5p ectopic expression and combined therapy on the apoptosis of A172 cells. Our results have shown that hsa-miR-34a-5p significantly increases the apoptotic rate in A172 cells (P-value = 0.0025) (Figure 9A, B(Fig. 9)). Also, hsa-miR-34a-5p significantly increases the temozolomide-mediated apoptosis on A172 cells (P-value = 0.0055) (Figure 9B(Fig. 9)). Consistent with this, hsa-miR-34a-5p significantly upregulates *CASP8* and *CASP9* expression and potentiates temozolomide-mediated *CASP8* and *CASP9* upregulation in A172 cells (all P-values < 0.0001) (Figure 9C, D(Fig. 9), respectively). However, hsa-miR-34a-5p significantly downregulates *CASP3* expression (P-values < 0.0001) (Figure 9E(Fig. 9)). Overall, hsa-miR-34a-5p increases the anti-tumoral effect of temozolomide on A172 cell apoptosis.

### hsa-miR-34a-5p ectopic expression increases the anti-tumoral effect of temozolomide on the A172 cell cycle

**Figure 10:**
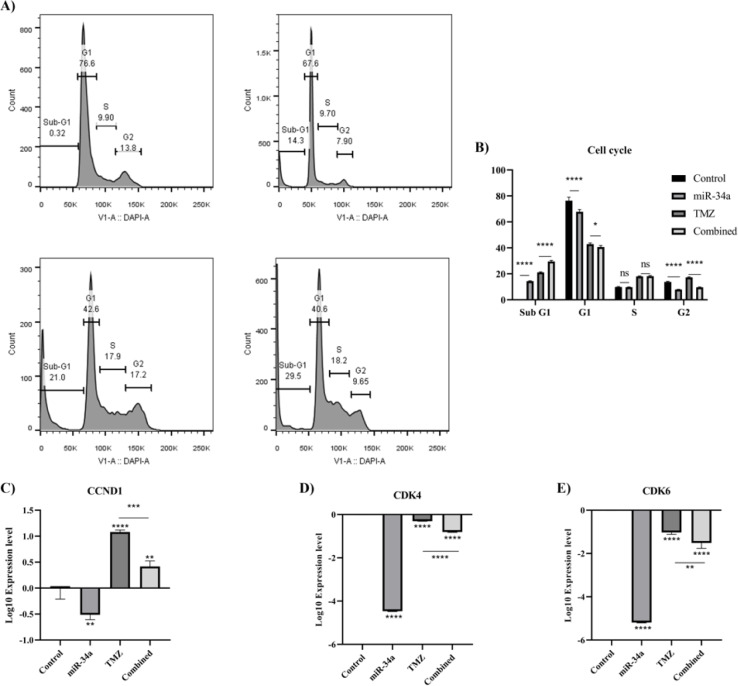
The effect of hsa-miR-34a-5p ectopic expression and temozolomide on the cell cycle distribution of A172 cells. A) The cell cycle distribution. The Y-axis denotes the distribution of cells in the cell cycle phases. B) The comparison of cell cycle distribution between groups. C) *CCND1* mRNA expression. D) *CDK4* mRNA expression. E) *CDK6* mRNA expression. ns: non-significant, *: P-value≤0.05, **: P-value≤0.01, ***: P-value≤0.001, and ****: P-value≤0.0001

Afterwards, we investigated the effect of hsa-miR-34a-5p increased expression and combined therapy on the cell cycle of A172 cells. It has been found that hsa-miR-34a-5p significantly arrests the cell cycle at the sub-G1 phase in A172 cells (P-value < 0.0001) (Figure 10A, B(Fig. 10)). Also, hsa-miR-34a-5p significantly increases the temozolomide-mediated cell cycle arrests at the sub-G1 of A172 cells (P-value < 0.0001) (Figure 10A, B(Fig. 10)). In line with these, hsa-miR-34a-5p significantly downregulates *CCND1* and *CDK4*, and *CDK6* expression in A172 cells (P-value = 0.0034, P-value < 0.0001, and P-value < 0.0001, respectively) (Figure 10C, D, E(Fig. 10)). Besides, hsa-miR-34a-5p potentiates temozolomide-mediated *CDK4* and *CDK6* downregulation in A172 cells (P-value < 0.0001 and P-value = 0.0085, respectively) (Figure 10D, E(Fig. 10)). Thus, hsa-miR-34a-5p enhances the anti-tumoral effect of temozolomide on the cell cycle of A172 cells.

### The combination therapy decreases the clonogenicity of A172 cells

**Figure 11:**
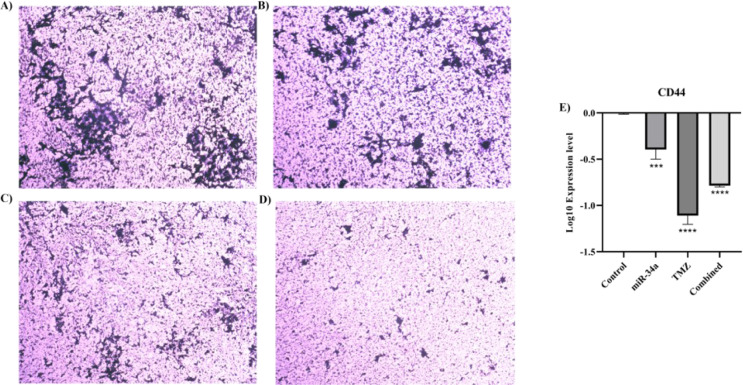
The effect of hsa-miR-34a-5p ectopic expression and temozolomide on the colony formation of A172 cells. A) Colony formation of the control group. B) Colony formation of hsa-miR-34a-5p. C) Colony formation of temozolomide. D) Colony formation of the combined group. E)* CD44* mRNA expression. ***: P-value≤0.001 and ****: P-value≤0.0001

We performed the colony formation assay to assess the clonogenicity and stemness of A172 cells. Our results have shown that hsa-miR-34a-5p decreases the clonogenicity of A172 cells (Figure 11B(Fig. 11)). Also, the combined therapy has substantially decreased the clonogenicity of A172 cells (Figure 11D(Fig. 11)). Consistent with our results, hsa-miR-34a-5p and combined therapy significantly downregulate *CD44* mRNA expression (P-value = 0.0006 and P-value < 0.0001, respectively) (Figure 11E(Fig. 11)). 

### hsa-miR-34a-5p, temozolomide, and the MAPK pathway in A172 cells

**Figure 12:**
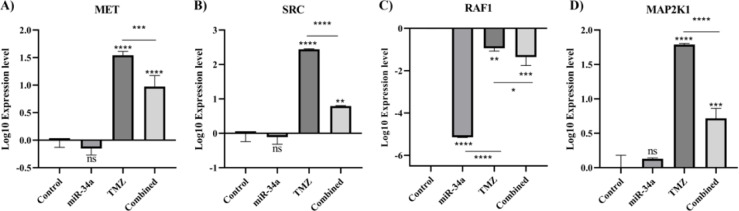
The effect of hsa-miR-34a-5p ectopic expression and temozolomide on the mRNA expression of singling factors in A172 cells. A) *MET* mRNA expression. B)* SRC* mRNA expression. C)* RAF1* mRNA expression. D)* MAP2K1* mRNA expression. ns: non-significant, *: P-value≤0.05, **: P-value≤0.01, ***: P-value≤0.001, and ****: P-value≤0.0001

Following identifying *RAF1* as the potential target of hsa-miR-34a-5p, we studied the MAPK pathway. It has been found that hsa-miR-34a-5p significantly downregulates *RAF1* expression in A172 cells and potentiates the inhibitory effect of temozolomide on *RAF1* expression (P-value < 0.0001 and P-value = 0.0012) (Figure 12C(Fig. 12)). Although hsa-miR-34a-5p does not significantly downregulate the expression of *MET*, *SRC*, and *MAP2K1*, the combination therapy significantly decreased the expression of *MET*, *SRC*, and *MAP2K1* compared to the temozolomide group (Figure 12A, B, D(Fig. 12)). Therefore, hsa-miR-34a-5p and the combination therapy can regulate the MAPK pathway compared to the control and temozolomide groups, respectively. 

## Discussion

Although substantial advances have been made in our understanding of GBM biology, the prognosis of affected patients is still unfavorable. Therefore, there is a pressing need to develop novel treatments for GBM. Based on our study, hsa-miR-34a-5p ectopic expression enhances the chemosensitivity of A172 cells to temozolomide and increases the anti-tumoral effect of temozolomide on the cell viability, migration, cell cycle, apoptosis, and clonogenicity of A172 cells. 

hsa-miR-34a-5p belongs to the miR-34a family that has anti-tumoral properties in different cancers (Li et al., 2014[21], 2021[23]; Noorolyai et al., 2022[32]). The miR-34 family members are the direct target of p53, and their upregulation has been associated with cell cycle arrest and apoptosis activation (Hermeking, 2010[17]). Low expression of miR-34a has been associated with poor overall survival and progression-free survival of patients with high-grade gliomas (Gao et al., 2013[13]). Li et al. have reported that hsa-miR-34a-5p is downregulated in human GBM tissues, and this miR inhibits the cell cycle progression, cell viability, proliferation, and invasion of GBM *in-vitro* and *in-vivo* via targeting c-MET in GBM cells (Li et al., 2009[24]). Duan et al. have shown that miR-34a targets BCL-2 expression, upregulates CASP3, CASP8, and CASP9, and stimulates the apoptosis of U87 cells (Duan et al., 2016[11]). It has been shown that miR-34a inhibits MMP9 expression and suppresses the migration of glioma. Besides, a MMP9 inhibitor has substantially attenuated the migration of U251 cells (Wang et al., 2019[41]). Consistent with our results, hsa-miR-34a-5p ectopic expression inhibits cell viability, arrests the cell cycle, increases apoptosis, decreases stemness, and suppresses the migration of A172 cells. Besides enhancing the chemosensitivity of A172 cells to temozolomide, the increased expression of hsa-miR-34a-5p potentiates the mentioned anti-tumoral effect of temozolomide on A172 cells. In terms of molecular mechanism, hsa-miR-34a-5p downregulates *MMP2*, *CDK4*, *CDK6*, *CCND1*, and *CD44* and upregulates *CASP9* and *CASP8* in A172 cells. In addition, the combined treatment has considerably decreased *CDK4*, *CDK6*, *CCND1*, and *MMP2* compared to monotherapy with temozolomide in A172 cells. However, monotherapy with hsa-miR-34a-5p and combined therapy increased PD-L1 expression (Supplementary Figure. 1). After observing these overall anti-tumoral effects, we studied the underlying singling pathway that can mediate these anti-tumoral effects. 

The Ras-Raf-MEK-ERK pathway has been implicated in various cancer development (Abdoli Shadbad et al., 2020[2]; Derakhshani et al., 2021[10]; Pearson and Regad, 2017[33]). Abel et al. have shown that the ectopic expression of KRas in murine glioneuronal precursor cells leads to intermediate and invasive glioma cells with 100 % penetrance (Abel et al., 2009[4]). Consistent with this, Holmen et al. have shown that suppression of KRas results in tumor regression and increased survival of GBM animal models, and re-introduction of KRas initiates tumor growth (Holmen and Williams, 2005[19]). Lyustikman et al. have reported that RAF1 is upregulated in human GBM tissues and RAF1 activation interacts with AKT to develop GBM; the histological feature of RAF1-induced GBM has been comparable to KRas-induced GBM (Lyustikman et al., 2008[28]). Besides, the combined upregulation of Ras and Akt transforms astrocytes and neural progenitors into GBM cells in mice (Holland et al., 2000[18]). Upon the activation of the tyrosine kinase receptors, like hepatocyte growth factor receptor (HGFR/c-MET), the cascade activation of the Ras-Raf-MEK-ERK signaling factors leads to cell proliferation and invasion (Mao et al., 2012[30]). The phosphorylation of tyrosines Y1349 and Y1356 on the c-MET activates the Ras-Raf-MEK-ERK pathway, leading to activation of the cell cycle, regulation of matrix metalloproteinase expression, and invasion in malignant cells (Abounader and Laterra, 2005[5]). It has been reported that the knockdown of the SF/HGF and c-Met has substantially decreased clonogenicity and tumorigenicity in GBM animal models (Abounader et al., 1999[6]). Petterson et al. have reported that c-MET protein expression is considerably upregulated in GBM compared to grade II glioma (Petterson et al., 2015[35]). Aside from being associated with progression-free survival of GBM patients, protein overexpression of c-MET has been significantly associated with poor overall survival of affected patients (HR = 2.761, 95 % CI = 1.214-6.280, P-value = 0.015). Also, increased protein expression of c-MET has been associated with invasion and multifocal presentation of GBM in initial MRI imaging, and c-MET expression has been positively associated with the protein expression of MMP2, MMP9, and c-Myc in GBM tissues (Kong et al., 2009[20]). Therefore, the HGFR/c-MET-mediated Ras-Raf-MEK-ERK pathway is a considerable oncogenic intracellular signaling pathway in GBM development. Our analysis of the GSE13276 dataset has shown that *RAF1* is substantially upregulated in GBM tissues compared to non-tumoral tissues, and the TCGA-GTEx dataset has confirmed this upregulation. Since the tumor microenvironment comprises various types of cells, like malignant cells, tumor-infiltrating immune cells, and endothelial cells, we performed single-cell RNA sequencing to identify which cell type is responsible for *RAF1* upregulation. It has been found that *RAF1* is expressed in astrocytes. Following the identification of *RAF1* upregulation in GBM tissues and astrocytes, our *in-silico *studies have demonstrated that *RAF1* might be the direct target of hsa-miR-34a-5p and there is a significant negative correlation between *RAF1* and hsa-miR-34a-5p in GBM tissues. Therefore, the effect of hsa-miR-34a-5p on GBM development and the c-MET/MAPK pathway was studied.

The current study has several strengths. First, this study had comprehensive *in-silico* analyses. Second, we studied the effect of hsa-miR-34a-5p not only on the characteristics of GBM cells but also on the molecular mechanism and signaling pathways derived from *in-silico* results. However, this study suffers from some limitations as well. First, we could not include *in-vivo* and *ex-vivo* studies. Second, the protein expression of studied signaling factors could not be studied. Third, only one cell line of GBM has been studied. Collectively, hsa-miR-34a-5p targets *RAF1* and potentiates the temozolomide anti-tumoral effect on A172 cells.

## Conclusion

hsa-miR-34a-5p targets *RAF1* expression, and its combination with temozolomide suppresses the MAPK pathway compared to temozolomide; hsa-miR-34a-5p has considerable anti-tumoral effects on the cell viability, migration, cell cycle, apoptosis, and clonogenicity of A172 cells. Besides, this miR improves the chemosensitivity of A172 cells and increases the anti-tumoral effect of temozolomide on A172 cells.

## Declaration

### Conflict of interest 

The authors declare that they have no conflict of interest.

### Acknowledgments

The research protocol was approved & supported by Student Research Committee, Tabriz University of Medical Sciences (grant number: 67581). This study was also approved by the Ethics Committee of Tabriz University of Medical Sciences (IR.TBZMED.VCR.REC.1400.059).

### Author contributions

MAS: conceptualization, investigation, methodology, writing - original draft, software, validation, and formal analysis. AB: methodology, investigation, and validation. BB: supervision and editing.

## Supplementary Material

Supplementary information
