# Supplementary material for: hsa-miR-34a-5p enhances temozolomide anti-tumoral effects on glioblastoma: in-silico and in-vitro study
Source: EXCLI J. 2024 Mar 13;23:384–400. doi: 10.17179/excli2023-6404 (PMC11036064; doi:10.17179/excli2023-6404)
Supplement: Supplementary information [file EXCLI-23-384-s-001.pdf]

EXCLI Journal 2024;23:384-400 – ISSN 1611-2156
Received: July 26, 2023, accepted: March 04, 2024, published: March 13, 2024

Supplementary information to:

Original article:

HSA-MIR-34A-5P ENHANCES TEMOZOLOMIDE ANTI-TUMORAL EFFECTS ON GLIOBLASTOMA: *IN-SILICO* AND *IN-VITRO* STUDY

Mahdi Abdoli Shadbad[1,2], Amir Baghbanzadeh[2], Behzad Baradaran[2,3*]

[1] Student Research Committee, Tabriz University of Medical Sciences, Tabriz, Iran
[2] Immunology Research Center, Tabriz University of Medical Sciences, Tabriz, Iran
[3] Department of Immunology, Faculty of Medicine, Tabriz University of Medical Sciences, Tabriz, Iran

* **Corresponding author:** Behzad Baradaran, Ph.D., Professor of Immunology, Immunology Research Center, Tabriz University of Medical Sciences, Tabriz, Iran, Daneshgah St, Tabriz, Iran. Phone number: +98 914 403 0526, E-mail: baradaranb@tbzmed.ac.ir

https://dx.doi.org/10.17179/excli2023-6404

This is an Open Access article distributed under the terms of the Creative Commons Attribution License (http://creativecommons.org/licenses/by/4.0/).

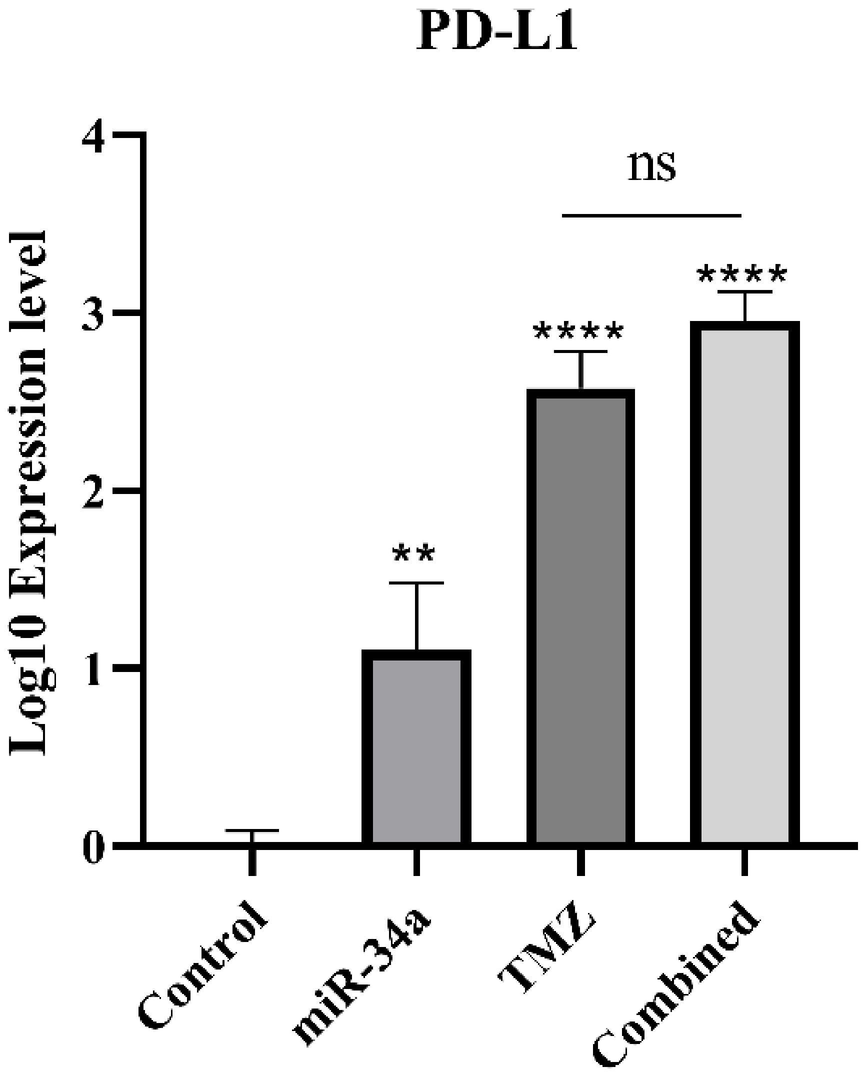

Supplementary Figure 1: The effect of miR-34a, temozolomide, and combination therapy on the PD-L1 expression in A172 cells
